# Nutritional Biomarkers in Children and Adolescents with Beta-Thalassemia-Major: An Egyptian Center Experience

**DOI:** 10.1155/2014/261761

**Published:** 2014-04-08

**Authors:** Laila M. Sherief, Sanaa M. Abd El-Salam, Naglaa M. Kamal, Osama El safy, Mohamed A. A. Almalky, Seham F. Azab, Hemat M. Morsy, Amal F. Gharieb

**Affiliations:** ^1^Department of Pediatrics, Zagazig University, Egypt; ^2^Department of Pediatrics, Faculty of Medicine, Cairo University, Cairo, Egypt; ^3^Department of Biochemistry, Zagazig University, Egypt

## Abstract

*Background and Aim*. Trace elements and vitamins play a vital role in human body to perform its function properly. Thalassemic patients are at risk of micronutrient deficiency. This study estimated levels of vitamins A, C, E, B12, folic acid, total homocysteine (tHcy), and methylmalonic acid (MMA) along with trace elements, zinc, copper, and selenium in Beta-thalassemia-major patients. *Methods*. This study included 108 patients with Beta-thalassemia-major and 60 age and sex matched healthy children. Serum levels of vitamin A, E, C, tHcy, and MMA were estimated by high pressure liquid chromatography while serum levels of folic acid and B12 were estimated by thin layer chromatography. Serum zinc, copper, and selenium were determined by atomic absorption spectrometry. *Results*. There was a significant decrease of vitamins A, C, E, and B12 and trace elements zinc, copper, and selenium in thalassemic patients as compared to controls. tHcy and MMA were significantly elevated in patients. No significant correlations were found between the serum levels of the studied vitamins and trace elements as regards age, frequency of transfusion, duration of transfusion, and serum ferritin. *Conclusion*. The level of various nutritional biomarkers (vitamins A, C, E, and B12 and trace elements zinc, copper, selenium) was reduced in chronically transfused Egyptian thalassemic patient. These patients should have periodic nutritional evaluation and supplementation. Multicenter studies are highly recommended.

## 1. Introduction

Hemoglobin disorders are the most common clinically serious single gene disorders in the world. “In Egypt beta-thalassemia-major is the most common type with carrier rate of 5.3 to ≥9% and 1000 new cases born with beta-thalassemia-major per 1.5 million live births per year” [1]. Thalassemic patients have ineffective erythropoiesis and accelerated red cell turnover owing to the short life span of red blood cell [2] which results in increased body demand of energy and nutrients to maintain normal erythropoiesis [3, 4]. Nutritional status is becoming increasingly important. Patients with thalassemia commonly exhibit inadequate growth, poor immune function, decreased bone mineralization, increased oxidative stress, and all morbidities which link to poor nutritional status [5–7].

Vitamins and trace minerals represent key buffers against oxidative damage. However, chronic demands on oxidative buffering capacity may produce conditional deficiencies in key amino acids and enzymatic cofactors [8]. Although isolated vitamins deficiency has been reported in B-thalassemia-major, there are still limited studies on comprehensive evaluation of vitamins and trace minerals in chronically transfused B-thalassemia-major.

The aim of the present study was to determine the serum levels of vitamins (A, C, E, folic acid, and B12) and trace elements (zinc, copper, and selenium) in chronically transfused thalassemic patients and their relation to age and iron burden.

## 2. Subjects and Methods

This cross sectional study was carried on 108 Egyptian children and adolescents with B-thalassemia-major recruited from those attending the Pediatric Hematology Outpatient Clinic and Inpatient Ward, Pediatric Hospital, Zagazig University, Egypt, during the period from January 2012 to January 2014, fulfilling the following inclusion and exclusion criteria.

Inclusion criteria:

1. age: less than 18 years old,
2. sex: both males and females.

Exclusion criteria:

1. patients with serological evidence of hepatitis B, C, delta-virus, or human immunodeficiency virus infections,
2. associated diabetes or thyroid dysfunction,
3. patients with any underlying metabolic, autoimmune, or systemic diseases other than B-thalassemia-major.

Sixty age and sex matched healthy subjects from those attending the well child clinics served as controls.

Written informed consent was obtained from all the guardians of patients and controls for the agreement for participation of their children in the study. The study was approved by the research and ethical committees of Zagazig University of Medical Sciences, Egypt.

All included patients were subject to detailed history taking and thorough clinical examination with special emphasis on the disease duration, transfusion history, chelation therapy details, renal, hepatic, and nutritional histories, and history of splenectomy, along with anthropometric measurements.

The following laboratory investigations were taken while the patients and controls were fasting and just before packed RBCs transfusion for the patients, including complete blood count (CBC) using Sysmex XT-1800i (Sysmex, Japan), examination of Leishman-stained smears for red blood cell (RBCs) morphology and differential white blood cells (WBCs), peripheral blood staining by Brilliant Cresyl blue and examination of stained smear for reticulocyte count, qualitative and quantitative hemoglobin analysis using HPLC by D-10 (BioRad, Marnes La Coquette, France), and liver and kidney function tests as well as serum ferritin on Cobas Integra 800 (Roche Diagnostics, Mannheim, Germany). Serum ferritin level was measured every 3 months during the study with calculation of the mean value of the last year prior to the study in order to know the ferritin trend. Serum vitamin A (retinol) and vitamin E (alpha-tocopherol) were estimated by high pressure liquid chromatography (HPLC) according to the method of Tee and Khor [9]. Vitamin C (ascorbic acid) was measured by HPLC according to the method of Gazdik et al. [10].

Serum folic acid and B12 were estimated by Thin Layer Chromatography according to the method of Ponder et al. [11]. Serum total homocysteine is elevated in both folate and B12 deficiencies. Methylmalonic acid is a specific marker of B12 metabolic insufficiency. The serum levels of both tHcy and (MMA) were estimated by HPLC.

Serum trace elements were estimated by atomic absorption spectrometry according to method by Carrero and Tyson [12].

## 3. Statistical Analysis

Analysis of data was done using statistical program for Social Science version 15 (SPSS Inc., Chicago, IL, USA). Quantitative (numerical) variable was described in the form of ranges, means, and standard deviations. Qualitative (categorical) variables were described as numbers and percentages. In order to compare parametric quantitative variables between two groups, student *t*-test was applied or Mann-Whitney (MW) for small sample size. Comparison between categorical variables was performed using Chi-square (**χ** ^2^) test or Fischer's exact test, for small sample size. A *P* value <0.05 was considered significant in all analyses.

## 4. Results

The present study included 108 B-thalassemia-major patients (68 males and 40 females), with a mean age of 9.85 + 4.3 years (range; 2–17 years). Sixty healthy controls were included (36 males and 24 females) with a mean age 10 + 3.9 years (range; 4–17 years).  Table 1 shows the demographic, clinical, and laboratory data of the patients and controls. Patients had significantly lower weight and height as compared to controls. Positive consanguinity and thalassemic brothers or sisters in the family were detected in 62.05% and 39.8% of patients, respectively, as in our community there is high incidence of consanguineous marriage. All patients were transfusion dependent on packed RBCs transfusion index of 271.5 ± 54.6 mL/kg/year. They received one to three times transfusions per month of about 15 mL packed RBC/kg, aiming to keep their hemoglobin level not less than 9 g/dL. Patients were on regular folic acid supplementation of one mg per day. Thirty-one patients were splenectomized. The most common signs of vitamin deficiency were skin manifestations as xerodrema with scaling which were detected in 37 patients (34.3%) mainly related to deficiency of vitamin A, vitamin D, zinc, and fatty acids, in addition to dehydration.

Neuropsychiatric manifestations in the form of fatigue, depression, cognitive changes, numbness, and parathesia in the lower limbs were detected in 43 patients (39.8%), which were related to vitamin B12 deficiency, chronic anemia, hypoxia, iron overload, thromboemboli, use of deferoxamine, and the effect of the chronic nature of the disease itself.

Unavailability of the data concerning dietary regimens was the main limitation of the current study. Regarding chelation, most of patients were on combined therapy of deferoxamine and deferiprone (45 patients), 27 patients were on deferoxamine, and 36 patients were on deferiprone with only 43.5% compliance. Compliance was defined as receiving 75% or more of the prescribed chelating agent dose. We have the limitation of being dependent on history for compliance assessment which is not reliably accurate. Regarding the hematological and biochemical results of the studied patients and controls, there was a significant decrease of hemoglobin concentration and significant increase of total bilirubin, AST, ALT, and serum ferritin in thalassemic patients as compared to controls.

Table 2 summarizes the mean values and percentages of micronutrients in B-thalassemia-major patients compared to controls. Serum levels of retinol, ascorbic acid, *α*-tocopherol, B12, zinc, copper, and selenium were significantly lower while serum folic acid level was significantly higher in thalassemic patients compared to controls.

Serum level of tHcy was 9.57 ± 0.23 micromol/L in patients compared to 8.61 ± 0.49 micromol/L in controls with *P* value of 0.00. Serum level of MMA was 310.73 ± 19.18 nmol/L in patients compared to 260.21 ± 20.34 nmol/L in controls with *P* value of 0.00. The significant elevation of both serum tHcy and MMA further proved B12 deficiency.

**Figure 1:**
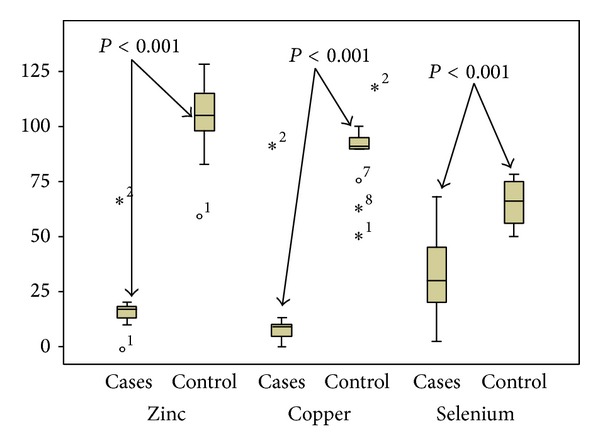
Comparison of serum level of traces elements in thalassemic patients compared to control.

The distribution of these trace elements with comparison of their levels in thalassemic patients and controls is shown in Figure 1.

No significant correlation was found between the serum levels of the studied micronutrients and the duration of blood transfusion, chelation therapy, serum ferritin, or age.

## 5. Discussion

Trace elements and the minerals play a vital role in the body to perform its functions properly. They should be present in the body in appropriate amounts and must be available for reacting with other elements to form critical molecules as well as to participate in various important chemical reactions [13].

*Zinc* is one of the essential micronutrients in human and considered as the most important mineral preceded by iron. It acts as a cofactor for more than 300 enzymes [14] and is considered a particularly important mineral to transfused patients with thalassemia because it is similar enough in size and charge to iron; therefore, it has potential to be chelated along with iron in these patients while being treated for iron overload. Tabatabei et al. [15] reported that 84% of B-thalassemia-major patients had zinc deficiency. They emphasized that the cause of zinc deficiency in these patients was due to insufficient zinc of dietary intake [15]. Similar reports were provided by other researchers [14, 16–20]. They recommended zinc supplements for thalassemic patients [14]. On the other hand, Mehdizadeh et al. [21] have reported significantly higher mean serum zinc level in thalassemic patients and commented that zinc deficiency is rare in thalassemia. Reshadat et al. [22] and Kosarian et al. [23] found that serum zinc level in their thalassemic patients and controls was within normal limits [22, 23]. They emphasized that medical treatment of those patients is not appropriate, so the value of zinc administration should be more evaluated [22].

The present study showed significantly lower level of zinc in thalassemic group than controls. The causes of zinc deficiency may be related to insufficient amount of zinc in daily meals, chronic hemolysis, and deferoxamine and deferiprone therapy [14].

*Copper* is one of the essential micronutrients of human body. This trace element acts as the cofactor for at least 30 enzymes [24]. It bears important antioxidant properties. It is a central component of the antioxidant superoxide dismutase molecule and also helps in the formation of a protein called ceruloplasmin thereby protecting the cells from free radical injury [13]. Some studies showed that there was an increase in serum level of copper in patients with B-thalassemia-major [18, 25–27]. Al-Samarrai et al. [20] concluded that the etiology of hypercupremia is hemochromatosis, which is a principal complication of thalassemia. On contrary, some other reports revealed reduction in serum level of copper [15, 28, 29]. Kassab-Chekir et al. [17] showed no change in serum copper concentration. These contradictory results may be explained by the fact that serum concentration of copper in patients with B-thalassemia-major depends on several factors including the amount of copper intake in daily diet, intestinal uptake of copper, iron accumulation, kidney function, copper to zinc ratio, and administration of deferoxamine [26, 27]. Changes in these factors in different combinations will seriously affect serum copper. Unfortunately our thalassemic patients had copper deficiency, indicating that the factors that influence copper levels were not properly controlled.

*Selenium* is an essential trace element in human plasma. It is an essential constituent of the enzyme glutathione peroxidase and also incorporates in various important proteins such as hemoglobin and myoglobin [30]. The present study detected a significant decrease in the plasma concentration of selenium in thalassemic patients compared to controls which was in agreement with the study of Nasr et al. [19] which showed a significant decrease in plasma concentrations of the essential element selenium as well as decreased plasma activity of selenium-dependent antioxidant enzyme glutathione peroxidase.

The fat soluble* vitamins A and E* are important nonenzymatic antioxidants [29]. The present work revealed that both vitamins were significantly decreased in thalassemic children compared to controls which is in agreement with other published researches [18, 19, 31, 32]. The significantly low levels of vitamin A and E can be explained by that excessive iron fraction generates a lipid peroxidation process with subsequent consumption of antioxidants [32, 33]. These results were supported by those of Gerster 1999 [34] and De Luca et al. 1999 [35]. Oral treatment with vitamin E improves the antioxidant/oxidant balance in plasma and red blood cells and counteracts lipid peroxidation process in B-thalassemia-major [32, 33].

*Vitamin C* is a potent water-soluble antioxidant in human. However, vitamin C has a particular role in vitamin E recycling and some reports have found vitamin C deficiency in thalassemic patients [36]. Our study reported significantly low level of vitamin C in thalassemic patients than controls. Claster et al. [18] indicated that 40–75% of patients with thalassemia have vitamin C deficiency. Dissagabutre et al. [37] found that all of his B-thalassemia-major patients had vitamin C deficiency. Low dose vitamin C supplement (100 milligram/day) was given and the authors did not find an increase of oxidative products. The authors found some evidence that supplementation of vitamin C plus vitamin E had more benefit than supplementation of vitamin E alone [37].

Claster and his colleagues [18] measured the circulating levels of a variety of essential nutrients in a sample of 24 chronically transfused patients with thalassemia. They found surprisingly low levels of both water soluble and fat soluble vitamins [18]. Only a few nutrients did not appear to be reduced, which included* vitamin B12 and *γ* tocopherol* [18]. However, the current study revealed significant decrease of both fat and water soluble vitamins including vitamin B12.

Only serum level of* folic acid* was increased in the thalassemic group as regular folic acid supplementation was part of our protocol of management of B-thalassemia-major patients.

All B-thalassemia-major patients were chronically anaemic. Chronically anemic patients have increased cardiac output to maintain oxygen delivery [38]. This produces a mildly hypercatabolic state, increases resting energy expenditure, and causes chronic oxidative stress [39]. These findings could contribute to increased consumption of nutrients. Malabsorption could also play a role. B-thalassemia-major patients develop siderosis of the exocrine pancreas. In B-thalassemia-major, pancreatic iron negatively correlates with circulating pancreatic trypsin levels [40] and patients have been documented to have significant decrease in stool elastase [41]. While these observations could clearly contribute to mal-absorption of fat soluble vitamins, it does not explain the low levels of water soluble vitamins found in these patients. The patient with thalassemia may be at increased risk of nutritional deficiencies due to elevated nutrient requirements and inadequate intake.

The present study demonstrates that chronically transfused patients with B-thalassemia-major have significant deficiencies of various nutritional markers (vitamins A, C, E, and B12 and trace elements zinc, copper, and selenium) which could be attributed to inadequate intake in the face of increased demand, consumption, and excretion. Dietary supplementation with various vitamins and trace elements along with appropriate diet might represent a promising way to improve the quality of life of thalassemic patients. Thalassemic patients should have periodic nutritional evaluation and supplementation as necessary. Nutritional counseling should be offered to these patients.

We highly recommend multicenter studies to explore relationships between circulating levels of nutrients and various comorbidities.

## Figures and Tables

**Table 1:** Demographic, clinical, and laboratory data of patients and controls.

Parameter	Patients	Controls	*P*
Age (years); mean ± SD (range)	9.5 ± 8.5 (2–17)	10.7 ± 3.9 (4–17)	0.59
Sex; *n* (%)			0.68
Male	68 (63)	36 (60)	
Female	40 (37)	24 (40)	
Height in cm; mean ± SD	110.77 ± 20.51	128.42 ± 25.81	0.04*
Weight in kg; mean ± SD	22.87 ± 10.51	36.63 ± 17.36	0.01*
Positive consanguinity; *n* (%)	67 (62.05)	—	—
Similar condition in the family; *n* (%)	43 (39.8)	—	—
Packed RBCs transfusion index (mL/kg/year)	271.5 ± 54.6	—	—
Splenectomy, *n* (%)	31 (29.6)	—	—
Hypovitaminosis: *n* (%)			
Xerodrema with scaling	37 (34.3)	—	—
Neuropsychiatric manifestations	43 (39.8)	—	
Chelation therapy: *n* (%)		—	—
Deferiprone	36 (33.3)		
Deferoxamine	27 (25)		
Both	45 (41.7)		
Compliance to chelation therapy, *n* (%)	47 (43.5)	—	—
WBCs (10^3^/uL); mean ± SD (range)	11.5 ± 4.5 (4.7–20.5)	7.3 ± 2.1 (4–11.7)	0.08
HB (g/dL)	7.39 ± 1.9	10.1 ± 1.1	0.001*
PLT (10^3^/uL)	311.7 ± 101.5	310.2 ± 61.7	0.94
Total bilirubin (mg/dL)	1.6 ± 1.2	0.8 ± 0.2	0.02*
AST (U/L)	20.7 ± 11.1	11.5 ± 1.99	0.02*
ALT (U/L)	20.1 ± 12.1	11.3 ± 2	0.04*
Creatinine (mg/dL)	0.7 ± 0.1	0.7 ± 0.1	0.61
Ferritin (ng/mL)	2003 ± 1694.9	157 ± 96.2	0.001*

*n*: number; *significant statistically; RBCs: red blood cells; WBCs: white blood cells; HB: hemoglobin; PLT: platelets; AST: aspartate transaminase; ALT: alanine transaminase.

**Table 2:** Serum level of vitamins and trace elements in thalassemic patients.

Parameter	Cases	Control	*t*	*P*
Retinol (ug/dL)			14.1	<0.001*
Mean ± SD	17.6 ± 7.6	42.8 ± 7.8		
Range	2–32	35–56		
Ascorbic acid (mg/dL)			13.9	<0.001*
Mean ± SD	0.256 ± 0.09	1.04 ± 0.4		
Range	0.11–0.47	0.61–1.74		
Alpha-Tocopherol (mg/dL)			16.56	<0.001*
Mean ± SD	0.498 ± 0.6	10.6 ± 4.5		
Range	0.025–2.16	5.7–19.9		
Folic acid (ng/mL)			3.96	0.001*
Mean ± SD	30.3 ± 12.3	17.7 ± 2.4		
Range	4–59	12–20.4		
B12 (pg/mL)			26.85	<0.001*
Mean ± SD	33.3 ± 40.7	332.7 ± 136.4		
Range (median)	8–170 (21)	160–510 (300)		
Zinc (ug/dL)			31.6	<0.001*
Mean ± SD	17.8 ± 13	103.6 ± 10.8		
Range	0.1–60.1	60–128		
Copper (ug/dL)			24.8	<0.001*
Mean ± SD	12.2 ± 17	90.5 ± 25		
Range (median)	0.1–93 (5)	59–120 (95)		
Selenium (ug/L)			9.612	<0.001*
Mean ± SD	31.5 ± 19.1	65.9 ± 6.3		
Range	2.4–68.2	56–74		

*Significant statistically.
